# Porous polymers bearing functional quaternary ammonium salts as efficient solid catalysts for the fixation of CO_2_ into cyclic carbonates

**DOI:** 10.1186/s11671-016-1529-z

**Published:** 2016-07-01

**Authors:** Sheng Cai, Dongliang Zhu, Yan Zou, Jing Zhao

**Affiliations:** State Key Laboratory of Coordination Chemistry, Institute of Chemistry and Biomedical Sciences, School of Chemistry and Chemical Engineering, Collaborative Innovation Center of Chemistry for Life Sciences, Nanjing University, Nanjing, 210093 China; Research and Development Centre, China Tobacco Anhui Industrial Co., Ltd., 9 Tianda Road, Hefei, Anhui 230088 China

**Keywords:** Ionic polymer, Mesoporous materials, CO_2_ fixation, Cycloaddition, Solid catalyst

## Abstract

A series of porous polymers bearing functional quaternary ammonium salts were solvothermally synthesized through the free radical copolymerization of divinylbenzene (DVB) and functionalized quaternary ammonium salts. The obtained polymers feature highly cross-linked matrices, large surface areas, and abundant halogen anions. These polymers were evaluated as heterogeneous catalysts for the synthesis of cyclic carbonates from epoxides and CO_2_ in the absence of co-catalysts and solvents. The results revealed that the synergistic effect between the functional hydroxyl groups and the halide anion Br^−^ afforded excellent catalytic activity to cyclic carbonates. In addition, the catalyst can be easily recovered and reused for at least five cycles without significant loss in activity.

## Background

Global climate change and excessive CO_2_ emission have attracted widespread public concern in recent years. Since CO_2_ is expected to be a highly abundant, quite cheap, nontoxic, and nonflammable C1 resource in the organic synthesis, the capture and utilization of CO_2_ to produce higher-value relevant chemicals, such as polycarbonates and cyclic carbonates, are receiving rising attention [1, 2]. In this context, the conversion of CO_2_ to cyclic carbonates via epoxide substrates is demonstrated to be an atom-economical reaction, and the products can serve as excellent aprotic polar solvents as well as intermediates in the production of pharmaceuticals and fine chemicals [3–5]. In the past few decades, extensive efforts have been devoted to develop efficient catalysts for the synthesis of cyclic carbonates, including salen-metal complexes [6–8], quaternary ammonium/phosphonium salts [9, 10], ionic liquids (ILs) [11, 12], molecular sieves [13], metal-organic frameworks [14, 15], and so on. Among them, ILs have become a class of promising candidates owing to their unique features of high thermal stability, variety of structures available, and easy shaping [16–18]. To simplify the separation process and improve the reusability of ILs, more efforts have been devoted to the supported IL catalysts [19, 20], which usually suffers from tedious preparation process, large mass transfer resistance, and the leaching of IL active sites. Thus, new strategies for the preparation of efficiently heterogeneous IL catalysts for the synthesis of cyclic carbonates are highly desirable.

Nanoporous polymeric materials have attracted increasing attention due to their versatile and tunable structures, high surface area, and tunable surface chemistry, which allow potential applications in gas storage, explosive detection, drug release, and catalysis [21, 22]. Particularly, ionic porous polymers obtained by the polymerization of monomeric ILs or copolymerization of ILs with other monomers have been investigated as innovative solid catalysts or catalyst supports [23, 24]. A wide range of ionic porous polymers have been developed and have shown excellent catalytic performances in numerous organic synthetic reactions, among which, the cycloaddition of CO_2_ with epoxide is a hot topic [25]. For example, Wang et al. [26] used an ionothermal method to prepare a novel meso-macroporous hierarchical poly(ionic liquid), which was applied as highly efficient heterogeneous catalysts for the conversion of CO_2_ into cyclic carbonates at ambient pressure. Zhang et al. prepared imidazolium salt-modified porous hypercross-linked polymers and used them as highly efficient solid catalysts for synergistic CO_2_ capture and conversion [27]. However, the present systems still suffer from long reaction time, high CO_2_ pressure, or high reaction temperature. Moreover, most of the present ionic porous polymers are based on imidazolium ILs, while the quaternary ammonium salt IL-based ionic porous polymers are scarce, although quaternary ammonium salts have been proved to be one of the most efficient catalysts for CO_2_ fixation. In addition, it has been reported that the functional groups such as hydroxyl and carboxyl are favorable for the cycloaddition reaction due to the synergistic effect with Br ions [28, 29]. These considerations prompted us to design new hydroxyl-containing quaternary ammonium salt-based ionic porous polymers as “task-specific” catalysts for the cycloaddition of CO_2_ with epoxides.

**Scheme 1:**
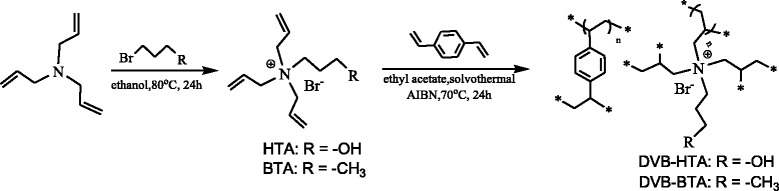
Synthesis of quaternary ammonium salt-based ionic porous polymers

Herein, we designed and synthesized a new type of hydroxyl functionalized quaternary ammonium salt-based ionic porous polymer via the radical copolymerization of hydroxyl functionalized triallylamine and divinylbenzene (DVB) (Scheme 1). The resulting porous materials with very high surface area can be sufficiently applied for the production of cyclic carbonates from CO_2_ and epoxides, showing high conversion and selectivity, easy recovery, and steady reusability.

## Methods

All chemicals in this work were used as received without further purification. Triallylamine (TAA); divinylbenzene (DVB), 3-bromo-1-propanol, 1-bromobutane, epichlorohydrin (ECH), and propylene oxide (PO) were purchased from Sigma Aldrich Reagent Co., LLC. Styrene oxide (SO), allyl glycidyl ether (AGE), and cyclohexene oxide (CHO) were provided by Aladdin Chemical Reagent Co., Ltd. Other reagents were laboratory-grade reagents from local suppliers.

### Characterizations

^1^H NMR spectra were collected on a Varian Mercury plus 400-MHz spectrophotometer at ambient temperature using D_2_O as solvent. CHN elemental analysis was performed on a vario EL cube elemental analyzer. Fourier transform infrared spectroscopy (FT-IR) spectra were recorded on a FT-IR instrument (Nicolet 360, KBr discs) in the 4000–400 cm^−1^ region. Thermogravimetric (TG) analysis was carried out with a STA409 instrument in nitrogen at a heating rate of 15 °C min^−1^. The nitrogen adsorption-desorption isotherms were measured on a BELSORP-MINI instrument at liquid nitrogen (77 K) temperature. The samples were evacuated at 423 K for 1.5 h before measuring. The specific surface areas were evaluated using the Brunauer-Emmett-Teller (BET) method, and the pore distribution was calculated by the BJH method from adsorption branches of isotherms. Transmission electron microscopy (TEM) analysis was performed on a JEM-2100 (JEOL) electron microscope operating at 200 kV. Scanning electron microscopy (SEM) images were recorded on a SUPERSCAN SSX-550 electron microscope (Shimadzu, Japan) operating at 20 kV. The morphology and the bromine (Br) element distribution were characterized by a Hitachi S-4800 field emission scanning electron microscope accompanied by energy-dispersive X-ray spectrometry.

### Synthesis of catalysts

#### Synthesis of HTA and BTA

Triallylamine (20 mmol, 2.74 g) and 3-bromo-propanol (20 mmol, 2.78 g) were dissolved in ethanol (15 mL). The mixture was then stirred at 80 °C for 24 h under nitrogen atmosphere. On completion, the solvent was removed by distillation and the solid product was washed with ethyl acetate three times to remove the unreacted substrates. After drying under vacuum, hydroxypropyl functionalized triallylamine (HTA) was obtained. ^1^H NMR (400 MHz, D_2_O, TMS) *δ*(ppm) = 1.93 (t, 2H, CH_2_), 3.21 (m, 2H, CH_2_), 3.51 (m, 2H, CH_2_), 3.62 (d, 6H, CH_2_), 5.81 (m, 6H, CH_2_), 5.94 (d, 3H, CH). The butyl functionalized triallylamine (BTA) was prepared accordingly in the same way and then was characterized by ^1^H NMR (400 MHz, D_2_O, TMS) *δ*(ppm) = ^1^H NMR (400 MHz, D_2_O, TMS) *δ*(ppm) = 0.86 (t, 3H, CH_3_), 1.26 (m, 2H, CH_2_), 1.69 (m, 2H, CH_2_), 3.13 (t, 2H, CH_2_), 3.72 (d, 6H, CH_2_), 5.59 (d, 6H, CH_2_), 5.90 (m, 3H, CH).

#### Synthesis of ionic porous polymers DVB-HTA and DVB-BTA

DVB (1.3 g, 10 mmol) and HTA (0.58 g, 2 mmol) were dissolved in 20 mL ethyl acetate and 4 mL methanol, respectively. These two solutions were mixed, and azodisisobutyronitrile AIBN (0.05 g) was added into it. After stirring at room temperature for 3 h, the mixture was solvothermally treated at 70 °C for 24 h. The yellow solid product DVB-HTA was filtered, washed with methanol three times, and dried under vacuum at 50 °C for 24 h. CHN elemental analysis for DVB-HTA found the following (wt.%): C 86.87, H 8.64, N 1.07. The ionic porous polymer DVB-BTA was prepared in the same way by reacting DVB with BTA. CHN elemental analysis for DVB-BTA found the following (wt.%): C 87.73, H 8.91, N 1.13.

#### Typical procedure for cycloadditions

As a typical example, ECH (20 mmol) and catalyst DVB-HTA-Br (0.05 g) were added into a 50-mL stainless steel autoclave equipped with a magnetic stirrer. After the reaction mixture was heated to 120 °C, CO_2_ was then charged into the reactor until the desired pressure of 1.2 MPa was reached. The reactor was cooled to ambient temperature after reacting 6 h, and the resulting mixture was filtered and the filtrate was analyzed by gas chromatography (GC) that was equipped with a FID and a DB-wax capillary column (SE-54 30 m × 0.32 mm × 0.25 μm). Biphenyl was used as an internal standard to calculate the catalytic conversion. GC-MS (SCIONSQ-456-GC) was used to analyze the purity and structure of the products. For the catalyst recycling, the filtered solid catalyst was directly used in the next run after washing with diethyl ether and drying.

## Results and discussion

### Preparation and characterization of catalysts

**Fig. 1:**
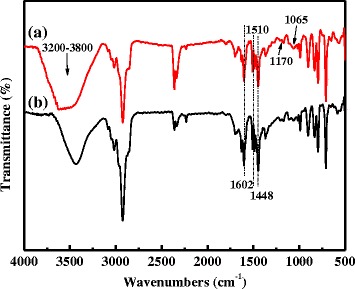
FT-IR spectra of *(a)* DVB-HTA and *(b)* DVB-BTA

The hydroxyl functionalized unsaturation quaternary ammonium salt (HTA) was first prepared by reacting 3-bromopropyl alcohol and triallylamine and verified by ^1^H NMR spectroscopy. Then, quaternary ammonium salt IL-based ionic porous polymer (DVB-HTA) was synthesized through the radical copolymerization of DVB with HTA (Scheme 1). The butyl functionalized quaternary ammonium salt-based ionic polymer DVB-BTA was prepared accordingly. The obtained samples were characterized by FT-IR analysis, and the spectra are shown in Fig. 1. DVB-HTA shows the characteristic bands of –OH (3200~3800 cm^−1^), C–O (1065 cm^−1^), and C–N stretching vibrations (1170 cm^−1^). The peaks approximately between 1448 and 1602 cm^−1^ can be attributed to the skeletal vibration of aromatic ring in DVB. The results indicate the successful copolymerization of HTA and DVB.

**Fig. 2:**
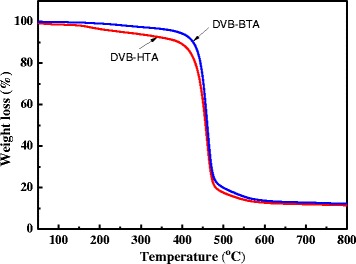
TG curves of DVB-HTA and DVB-BTA

The ionic polymers are white powders and insoluble in water or common organic solvents, such as acetone, ethanol, chloroform, hexanes, *N*,*N*-dimethylformamide (DMF), and tetrahydrofuran (THF). The CHN elemental analysis for these polymers reveals that the molar ratio of quaternary ammonium salts to DVB was about 1:9, as illustrated in Scheme 1. The TG profile in Fig. 2 shows that DVB-HTA and DVB-BTA are stable up to 400 °C. For DVB-HTA, the weight loss of nearly 4 % in the range of 200~400 °C may be due to the elimination of hydroxyl functional groups in HTA. The further weight loss in 400~600 °C was attributed to the decomposition of organic polymer framework.

**Fig. 3:**
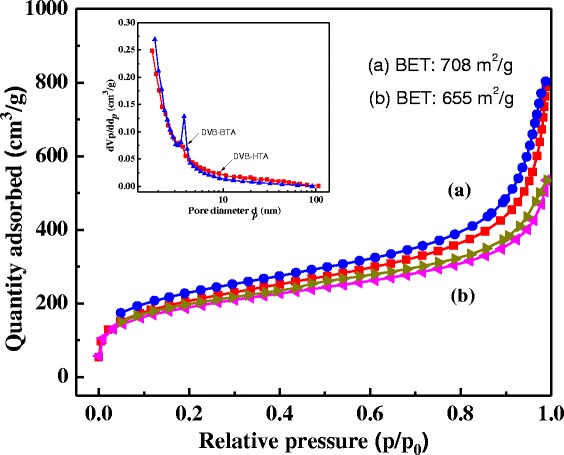
Nitrogen adsorption-desorption isotherms and pore size distributions of **a** DVB-HTA and **b** DVB-BTA

Figure 3 shows the N_2_ adsorption-desorption isotherms and the corresponding pore size distributions of DVB-HTA and DVB-BTA. They display typical IV-type isotherm with hysteresis loops at relative pressure of 0.8 < *P*/*P*_0_ < 1. Correspondingly, their average pore sizes are distributed at 5–7 nm. The Brunauer-Emmett-Teller (BET) surface areas and pore volumes of DVB-HTA and DVB-BTA are 708 and 655 m^2^/g and 1.21 and 0.83 cm^3^/g, respectively.

**Fig. 4:**
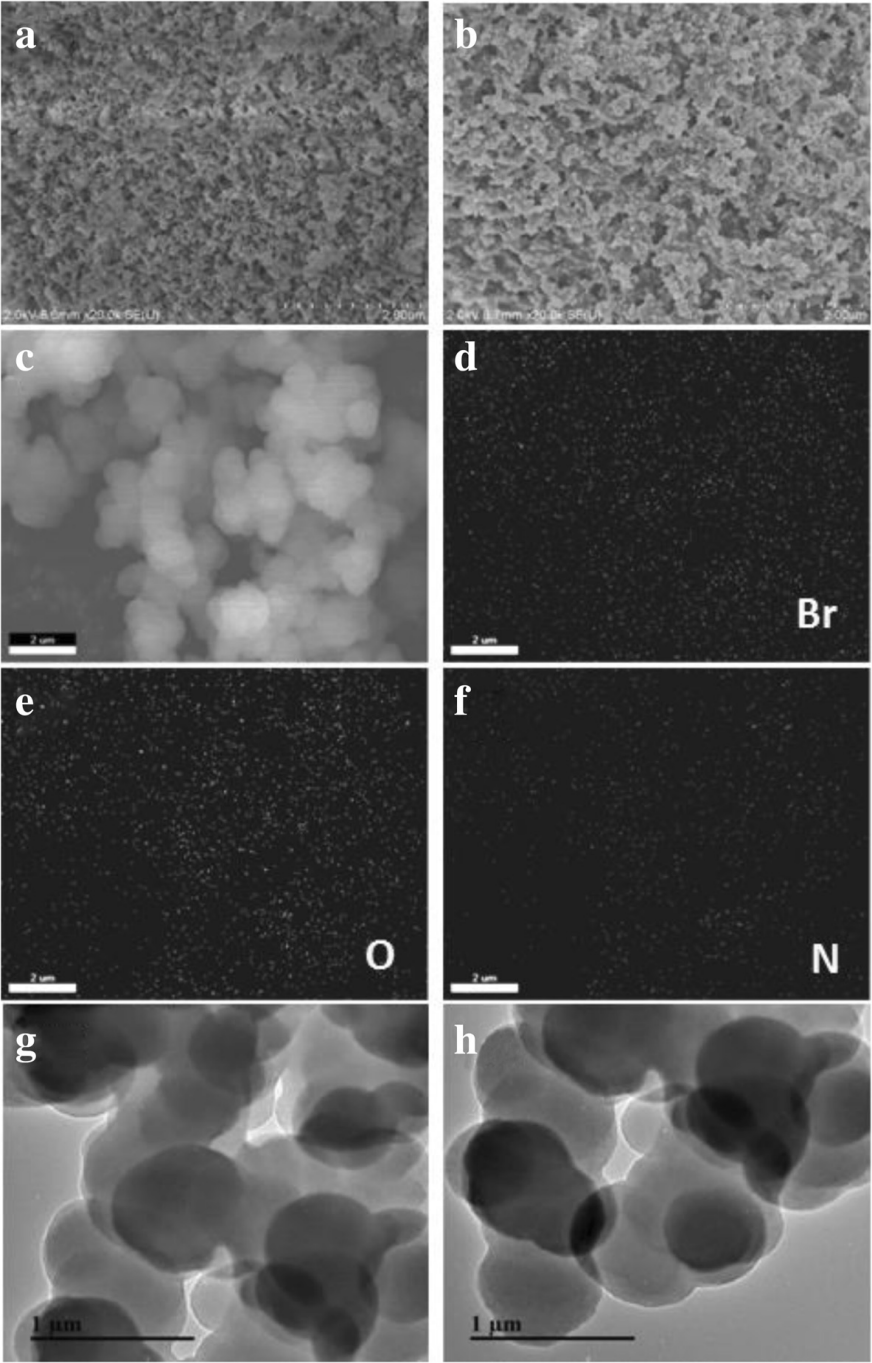
SEM images of **a**, **c** DVB-HTA and **b** DVB-BTA; EDS elemental mapping of **d** Br element, **e** O element, and **f** N element; and TEM images of **g** DVB-HTA and **h** DVB-BTA

The SEM images of DVB-HTA and DVB-BTA in Fig. 4a, b show the abundant wormhole-like morphology with micrometer size. The EDS structural characterization validates the uniform distribution of Br, O, and N on the surface of DVB-HTA, which further confirms the successful combination of HTA and DVB on one catalyst framework. In the TEM images of DVB-HTA and DVB-BTA in Fig. 4g, h, the porosity could be clearly observed. It is proposed that functional quaternary ammonium salt with three vinyl groups acts as the cross-linking node, which intertwines with the DVB to form a highly cross-linked network.

### Catalytic performance

The catalytic activities were first tested on epichlorohydrin (ECH) as the model substrate at 120 °C with 1.2 MPa CO_2_ under solvent-free conditions, and the results are listed in Table 1. No product was detected in the absence of the catalyst (entry 1). The ionic polymers are insoluble in the reaction system, and DVB-HTA bearing hydroxyl functional groups exhibited 90 % yield with 98 % selectivity (entry 2). It is higher than that of hydroxyl-free ionic polymer DVB-BTA, which gave 84 % yield with a relative low selectivity of 92 % (entry 3). The quaternary ammonium salt monomers HTA offered 84 % yield (entry 4) that is higher than that of hydroxyl-free BTA which showed 77 % yield (entry 5), but it is still lower than that of ionic polymer DVB-HTA. Additionally, HTA and BTA caused homogeneous catalysis, and the catalyst cannot be easily recovered and reused. The results of the above control experiments imply that the highly cross-linked polymer framework endows the catalyst with insolubility and thus result in heterogeneous catalysis. Although the halogen Br^−^ ions act as the active centers that are indispensable for the catalytic efficiency of the catalyst, the mesoporous framework with such high surface area can accelerate the mass transfer and allow the Br^−^ give full play as active centers, thus giving high catalytic activity. Furthermore, the hydroxyl functionalized samples demonstrated higher activity than the hydroxyl-free samples, demonstrating that the –OH groups are more favorable for the cycloaddition reaction mostly due to the synergistic effect with Br^−^ ions. This result is inconsistent with those of the previous reports [30, 31].

**Table 1:** Cycloaddition of CO_2_ and ECH catalyzed by various catalysts

Entry	Catalyst	Solubility	Yield^a^ (%)	Sel^b^ (%)
1	No catalyst	Homogeneous	–	–
2	DVB-HTA	Heterogeneous	90	98
3	DVB-BTA	Heterogeneous	84	92
4	HTA	Homogeneous	84	97
5	BTA	Homogeneous	77	95

Reaction conditions: ECH (16 mmol), CO_2_ (1.2 MPa), catalyst (0.035 mmol based on Br), 120 °C, 3 h

^a^The yield of cyclic carbonate product

^b^The selectivity for the cyclic carbonate product; the byproduct is 3-chloro-1,2-propanediol

**Fig. 5:**
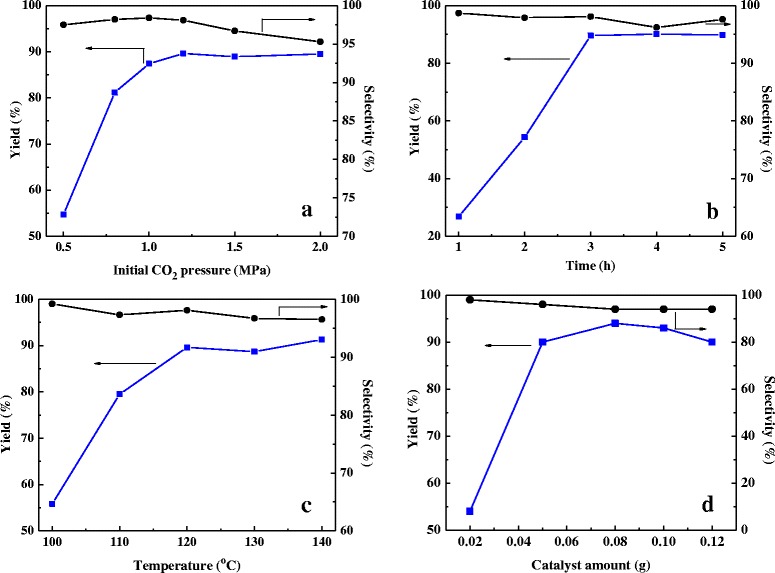
Influences of reaction conditions on the cycloaddition of CO_2_ and ECH over DVB-HTA. **a** Influence of initial CO_2_ pressure. **b** Influence of reaction time. **c** Influence of reaction temperature. **d** Influence of catalyst amount. Reaction conditions: catalyst 0.05 g, ECH 16 mmol, CO_2_ pressure 1.2 MPa, 120 °C, 3 h; for each figure, there is a specific parameter changed

The results of the experimental optimization in Fig. 5 show that the reaction conditions, including reaction time, CO_2_ pressure, the reaction temperature, and the catalyst amount, have remarkable influence on the yield of cyclic carbonate in the presence of DVB-HTA as a catalyst. The best reaction condition is 1.2 MPa CO_2_ pressure, 120 °C temperature, and 3-h reaction time. Notably, a further increase of the CO_2_ pressure exceeding 1.5 MPa or reaction temperature exceeding 130 °C led to a slight decrease of the cyclic carbonate yield, which may result from the further oxidation of the cyclic carbonate formed. For the catalyst amount, with the increasing the of the catalyst amount to 0.08 g, a high yield of 94 % with 98 % selectivity could be achieved, demonstrating the high efficiency of this novel catalyst DVB-HTA.

In order to investigate the scope of the ionic porous polymer DVB-HTA for the fixation of CO_2_, various functional-group-substituted epoxides, such as propylene oxide, styrene oxide, allyl glycidyl ether, and cyclohexene oxide, were studied as the substrates, and the result are summarized in Table 2. It can be seen that most of the epoxides can be transformed to the corresponding cyclic carbonates in high yield and selectivity at 120 °C with 1.2 MPa CO_2_ pressure and 0.05 g catalyst under solvent-free condition. In the case of cyclohexene oxide, a moderate yield of 56 % with 63 % selectivity was obtained. Notably, the coupling of CO_2_ with cyclohexene oxide is a rather difficult reaction, and it could not give a high yield of cyclic carbonate as well over other catalysts [32, 33]. This is possibly ascribed to the limited diffusion of large-sized epoxide substrate molecules into the pore canal of DVB-HTA thus exerting size-selective catalysis.

**Table 2:** Cycloaddition of CO_2_ with different epoxides catalyzed by DVB-HTA

Entry	Epoxide	Product	Time (h)	Con (%)^a^	Sel (%)^b^
1			3	90	98
2			6	93	99
3			6	89	98
4			4	83	98
5			12	56	63

Reaction conditions: epoxide (16 mmol), CO_2_ (1.2 MPa), catalyst DVB-HTA (0.05 g, 0.035 mmol), 120 °C

^a^The yield for the cyclic carbonate product

^b^The selectivity for the cyclic carbonate product, and the byproduct for entry 5 is mostly 1,2-hexanediol

**Fig. 6:**
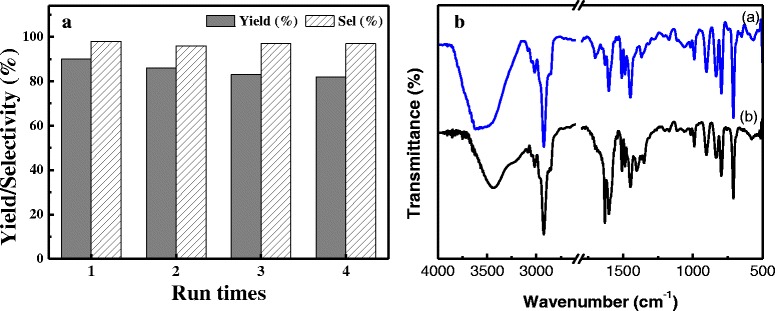
**A** Catalytic reusability of DVB-HTA for the cycloaddition of CO_2_ with ECH and **B** FT-IR spectra of *(a)* fresh DVB-HTA and *(b)* reused DVB-HTA. Reaction conditions: ECH 16 mmol, catalyst 0.05 g (0.035 mmol), 120 °C, CO_2_ 1.2 MPa, 3 h

The reusability studies of the catalyst DVB-HTA in the cycloaddition of CO_2_ with ECH have been tested. The results of a five-run recycling performance are shown in Fig. 6A. It is obvious that the yield of cyclic carbonate which is 90 % for the first run only marginally decreased down to 82 % for the fifth run. To check if structural deterioration occurred for the recovered catalyst, the recovered DVB-HTA was characterized by FT-IR. The IR spectrum of the reused DVB-HTA in Fig. 6B was very similar to that of the fresh one, demonstrating a durable catalyst structure accounting for the steady catalytic reuse. The weight loss in the operation for recovering the catalyst is usually unavoidable during the recycling tests. Thus, the very slow decrease of yield is mostly probably due to the weight loss in the operation for recovering the catalyst.

**Scheme 2:**
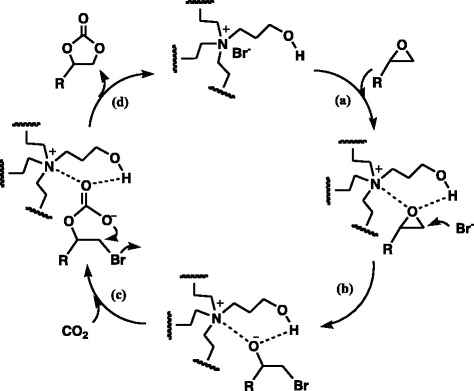
The proposed mechanism of the DVB-HTA-catalyzed fixation of CO_2_ with epoxides

According to previous reports, the cooperation of nucleophilic attack by a halogen anion and the activation by an onium cation through electron interaction could facilitate the opening of an epoxy ring [34, 35] and the functional groups such as the –OH and –COOH groups can also activate the cycloaddition reaction through hydrogen bonding [28, 29]. In this work, by rationalizing our experimental results and relating them to these numerous literatures, a synergistic catalysis of hydrogen bonding, electronic interaction, and nucleophilic attack for the cycloaddition of epoxides and CO_2_ to form cyclic carbonate was proposed, as illustrated in Scheme 2. The epoxy ring is first activated by the –OH group via a hydrogen bond interaction and the quaternary ammonium cations via electronic interaction (a). What follows is the opening of the epoxy ring through Br^−^ nucleophilic attack (b) then the insertion of CO_2_ into the transient species. Finally, the product cyclic carbonate was formed via an intramolecular nucleophilic attack, and the catalyst was regenerated to the original form (d). Notwithstanding, this mechanism is tentative and the detailed mechanistic studies to probe the intermediates during the cycloaddition reaction could be necessary, and research along this line will be conducted in the near future.

## Conclusions

In summary, we have developed a new type of quaternary ammonium salt-based ionic porous polymer, DVB-HTA, by the copolymerization of DVB and hydroxyl functionalized quaternary ammonium salts. The DVB-HTA was a highly cross-linked porous material with a large surface area of 708 m^2^/g and abundant Br^−^ anions and acted as efficient heterogeneous catalysts for the transformation of CO_2_ and epoxides into cyclic carbonates under metal-solvent-free conditions. The excellent catalytic performance of DVB-HTA results from the synergistic effect between the Br^−^ active centers and functional –OH groups. Moreover, the DVB-HTA had good recyclability, attributed to the durable highly cross-linked framework structure. This catalyst is potentially useful in industrial applications due to its low cost, excellent catalytic efficiency, and good stability.
